# The Clinical Impact of Patient Embarrassment in Gynecology: A Comprehensive Review of Barriers, Consequences, and Mitigation Strategies

**DOI:** 10.3390/medsci14020335

**Published:** 2026-06-22

**Authors:** Tudor Butureanu, Raluca Anca Balan, Ana-Maria Haliciu, Razvan Socolov, Demetra Socolov

**Affiliations:** 1 Department of Mother and Child, Grigore T Popa University of Medicine and Pharmacy, 700115 Iasi, Romania; raluca.balan@umfiasi.ro (R.A.B.); anna_cefalan@yahoo.com (A.-M.H.); socolov.razvan@gmail.com (R.S.); demetrasocolov@gmail.com (D.S.); 2 Department of Mother and Child, “Elena Doamna” University Hospital of Obstetrics and Gynecology, 700036 Iasi, Romania; 3 Pathology Department, “Elena Doamna” University Hospital of Obstetrics and Gynecology, 700398 Iasi, Romania

**Keywords:** patient embarrassment, shame, gynecological care, pelvic examination, doctor–patient communication, trauma-informed care, health literacy, cervical cancer screening, endometriosis, delayed diagnosis

## Abstract

Patient embarrassment represents a significant yet often underrecognized barrier to effective gynecological care. This review integrates multidisciplinary evidence from Embase, PubMed, PsycINFO, and the Cochrane Library (2000–2025) to examine the relationship between embarrassment, shame, and modesty and their impact on care-seeking behaviors, clinical outcomes, and healthcare utilization. Available data indicate that embarrassment is consistently associated with reduced participation in preventive screening, with up to one-third of non-attenders citing modesty-related concerns. In symptomatic patients, these emotional barriers contribute to clinically meaningful diagnostic delays, particularly in conditions such as cervical cancer, vulvar cancer, and endometriosis. Embarrassment also affects in-consultation behavior, with a substantial proportion of patients reporting withheld concerns or incomplete disclosure of medically relevant information. The consequences extend beyond delayed diagnosis to include reduced treatment adherence, increased disease severity at presentation, and higher healthcare costs due to more complex and resource-intensive interventions. Contributing factors include cultural stigma, prior negative clinical experiences, fear of judgment, and aspects of the clinical environment that may heighten patient vulnerability.

## 1. Introduction

Patient embarrassment and the closely related emotion of shame are not peripheral or trivial features of the clinical encounter; in many medical specialties they act as significant psychosocial barriers that compromise patient safety, diagnostic accuracy, and treatment outcomes [1,2]. Across general medicine, evidence suggests that emotional states such as shame, modesty, and the fear of negative judgment can alter patient behavior at every stage of the care continuum, motivating avoidance of necessary care, concealment of vital information, and non-adherence to prescribed treatments [1,2].

Gynecology occupies a particularly exposed position within this broader phenomenon. The gynecological consultation is an environment of inherent vulnerability, routinely requiring physical and psychological exposure of anatomical regions that are simultaneously medically significant and socially private [2]. For this reason, embarrassment in gynecological care is not a marginal patient experience; it is a clinically relevant determinant of outcomes. What begins as an internal emotional response can lead to measurable clinical consequences: delayed diagnosis of gynecological cancers—particularly cervical and vulvar cancer—and of endometriosis [3], reduced adherence to recommended treatment, and a progressive erosion of trust in the patient–provider relationship.

The clinical impact of patient embarrassment is substantial and well-documented. A significant portion of the patient population actively censors itself during medical consultations under these emotional pressures. In a recent French primary-care survey, nearly one-third (32.3%) of patients reported having left concerns untold during a consultation specifically because of embarrassment, modesty, or a fear of being judged by their clinician [4]. Inadequate doctor–patient communication has been implicated in up to 80% of all preventable adverse events (pAEs), which compromise patient safety and result from unsafe healthcare processes rather than the patient’s underlying medical condition; pAEs can occur across all medical specialties, including gynecology and obstetrics [5]. Obstetrical practice is particularly demanding due to high workloads, frequent emergencies (e.g., emergency cesarean sections), and the dual responsibility for both the mother and the fetus/newborn, which further amplifies the consequences of communication failure [5]. Some studies suggest that the phenomenon of withholding information is even more widespread, with up to 81.1% of patients admitting to having avoided disclosing at least one type of medically relevant information during a clinical encounter [4]. These findings indicate that embarrassment is not a marginal issue, but a common and clinically relevant barrier affecting care quality. The failure to recognize and address this emotional barrier is a failure to see the patient holistically, leading to a medical practice that may be technically proficient but emotionally and communicatively deficient, undermining its therapeutic goals.

## 2. Methods

### 2.1. Design

This work is a narrative review of multidisciplinary literature, undertaken to integrate quantitative and qualitative evidence on embarrassment-related barriers in gynecological care. A narrative design was chosen because the available evidence is heterogeneous in design (cross-sectional surveys, qualitative interviews, randomized trials of communication and environmental interventions, and clinical guidelines), drawn from clinical medicine, psychology, sociology, and public health, and does not lend itself to quantitative pooling. As discussed in the Limitations section, this design entails interpretive rather than meta-analytic conclusions.

### 2.2. Information Sources

Four major electronic databases were searched: PubMed, Embase, PsycINFO, and the Cochrane Library. Reference lists of included studies and of recent reviews on related topics were hand-searched to identify additional sources. The final search was completed in January 2026.

### 2.3. Search Strategy

The following Boolean string was used, adapted to the controlled vocabulary of each database (MeSH for PubMed/Cochrane; Emtree for Embase; APA Thesaurus for PsycINFO):

(“embarrassment” OR “shame” OR “modesty” OR “stigma”) AND (“gynecolog\” OR “obstetric\” OR “pelvic examination” OR “cervical screening” OR “Pap” OR “speculum”) AND (“patient” OR “women”) AND (“communication” OR “barriers” OR “help-seeking” OR “delay” OR “adherence”).

### 2.4. Date Range and Language

Records published between January 2000 and June 2025 were considered. Search results were restricted to publications in English, with the exception of one Polish-language study of Szymoniak et al., 2009 for which an English abstract was available and the numerical results could be independently verified.

### 2.5. Eligibility Criteria

Inclusion criteria:

- Original quantitative or qualitative studies, systematic reviews, narrative reviews, or clinical guidelines.
- Studies addressing (a) patient embarrassment, shame, or modesty in gynecological or closely related primary-care settings; (b) the impact of these emotional states on screening uptake, symptomatic presentation, in-consultation behavior, or treatment adherence; or (c) interventions intended to mitigate these emotional barriers.
- Adult and adolescent populations (≥13 years).

Exclusion criteria:

- Studies focused exclusively on provider embarrassment without patient-level data.
- Pediatric populations younger than 13 years.
- Conference abstracts without methodological detail.
- Editorials and opinion pieces without empirical or clearly delineated conceptual content.

### 2.6. Data Extraction and Thematic Synthesis

For each included study, the following information was extracted into a structured table: study design, country of conduct, population and sample size, clinical setting, outcome measures, and key findings. Findings were synthesized thematically into three domains:

1. **Barriers to access**—including procedural anxieties, communicative and topical triggers, and the fear of negative social evaluation; and how these affect uptake of screening and symptomatic presentation.
2. Clinical consequences—diagnostic delay, in-consultation information concealment, treatment non-adherence, and the resulting health and economic outcomes.
3. Mitigation strategies—patient-centered communication interventions, procedural and environmental modifications, and system-level training and education initiatives.

## 3. Limitations of the Review Design

Because this is a narrative review rather than a systematic review with a PRISMA-compliant flow diagram, the conclusions are interpretive rather than quantitatively pooled. The included studies are heterogeneous in design, population, and outcome measures, which limits the ability to draw direct comparisons or establish causality. Many studies rely on self-reported data, which is subject to recall and social desirability bias—particularly given the sensitive nature of the construct. Additional limitations are discussed in Section 4.

### 3.1. The Phenomenology of Embarrassment in the Gynecological Setting

Gynecological consultations can be particularly sensitive, and many patients report feelings of embarrassment or discomfort during these encounters [2]. Unlike many other medical specialties, it routinely involves the discussion of deeply personal topics and the physical examination of intimate parts of the body. This creates both physical and psychological exposure for the patient [2]. The pelvic examination, a cornerstone of gynecological practice, is frequently described in the literature as an “uncomfortable, painful, embarrassing, and anxiety-provoking experience” [6]. This single procedure places the patient in a physically vulnerable position—supine, with feet in stirrups—which can evoke a sense of powerlessness and loss of control [7]. This physical vulnerability is compounded by a psychological one. Patients often perceive their symptoms or diseases not as objective medical issues, but as personal defects, inadequacies, or shortcomings [2]. Concerns about cleanliness, vaginal odor, or sexual habits can become sources of intense self-consciousness and anxiety [7]. The patient is not just presenting a medical problem; she is presenting a part of herself that is deeply tied to identity, sexuality, and self-worth, making her acutely sensitive to the perceived or actual judgment of the clinician. The combination of physical exposure and emotional vulnerability makes these consultations especially likely to trigger feelings of shame, influencing every aspect of the clinical interaction.

### 3.2. Primary Triggers of Embarrassment and Shame

The general sense of vulnerability within the gynecological encounter is crystallized by several specific triggers that consistently emerge as sources of patient embarrassment and shame. Synthesizing the framework proposed by Hurtaud et al. [4] with the trigger taxonomy described by Balfe et al. [8], these can be broadly categorized into procedural anxieties, the discussion of sensitive topics, and the overarching fear of negative social evaluation.

The physical and procedural aspects of the examination are powerful triggers. The simple act of undressing is a significant source of discomfort for a large proportion of women, with over 40% reporting embarrassment related to this necessary step [9]. In a cross-sectional study by Yanikkerem and colleagues (*n* = 433 women attending a Turkish outpatient gynecology clinic), more than half of women (54.8%) reported feeling anxious or worried about their health during the pelvic examination, while 41.8% reported embarrassment about having to undress. A preference for a female doctor was expressed by 45.5% of women, whereas only 4.2% preferred a male doctor for obstetric and gynecological care; the remaining 49.9% indicated no preference. Most participants (62.1%) expected the doctor to explain their health condition following the examination, while 71.8% believed that the nurse should demonstrate understanding and gentleness, and 28.2% felt that the nurse should provide information about the pelvic examination [10].

The pelvic exam itself, involving the insertion of a speculum and the direct visualization of the genitalia, is cited as one of the most common anxiety-provoking medical procedures [11]. Women frequently experience anxiety and fear both before and during pelvic examinations. Identifying the underlying causes of these emotions is an essential first step toward reducing them. Across published studies, reported rates of anxiety or fear range from approximately 21% to 64% among women [6]. In a study by Yamikkerem and colleagues, more than half (54.8%) of 433 women attending an outpatient gynecology clinic reported feeling anxious or worried. Similarly, Phumdoung and colleagues found that 64% of women experienced anxiety and fear. Other studies have documented that up to 52% of women reported embarrassment, 21–49% reported anxiety, and 22–68% experienced pain during pelvic examinations [6]. In a large Nordic cross-sectional questionnaire study by Hilden et al. (*n* = 3641 women attending gynecological outpatient clinics across Sweden, Denmark, Norway, and Finland), univariate analyses revealed that perceived discomfort during gynecological examinations was significantly associated with younger age (18–25 years), student status, nulliparity, dissatisfaction with sexual life, a history of moderate or severe sexual abuse, negative emotional interactions with the examiner, and mental health conditions such as depression, anxiety, and insomnia. In contrast, having a male gynecologist and presenting with pain at the visit were not significantly related to discomfort [12]. These associations were reported in univariate analyses and should therefore be interpreted as descriptive rather than implying independent effects, as multivariable adjustment was not performed in the source study.

The physical sensations associated with the exam, such as the feeling of a cold instrument or a perceived lack of gentleness from the examiner, can amplify feelings of discomfort and violation [7]. These aspects of the procedure can intensify feelings of exposure and discomfort. Second, the topics of conversation inherent to gynecology are frequently laden with social taboos. Patients report extreme reluctance to discuss issues related to their sexual lives, including behavior, libido, and vaginal dryness, with these topics accounting for 15% of unvoiced concerns in the Hurtaud et al. study [4]. Other commonly cited embarrassing subjects include vaginal odor, abnormal discharge, urinary incontinence, and pain or other difficulties with intercourse [13]. Other commonly cited embarrassing subjects include vaginal odor, abnormal discharge, urinary incontinence, and pain or other difficulties with intercourse [13]. In a Polish hospital survey by Szymoniak et al., 70% of respondents reported that gynecological examinations are experienced as embarrassing and stressful; the most unpleasant aspect of a gynecological visit was the time spent in the examination chair (47%), followed by the preparation for the examination (30%). The examination itself was perceived as the least embarrassing part of the visit (21%). When specific procedures are considered, the vaginal examination was identified as the most embarrassing (40%), followed by rectal examination (33%), colposcopy (27%), and, to a much lesser extent, breast examination (2%) [14]. These topics are often intertwined with deeply ingrained social norms about hygiene, sexual responsibility, and personal privacy, making their disclosure feel like a transgression of social boundaries [8]. Finally, underlying both procedural and topical triggers is a fear of judgment and stigma. This fear of being evaluated negatively by the clinician is a driver of embarrassment and a key reason for withholding information [4]. The anxiety is particularly acute in situations where patients feel their behavior has violated social expectations, such as having unprotected sex and subsequently seeking STI testing [8]. The patient in this scenario feels shame not just about the potential for disease, but about the mistake that led her there, fearing the clinician’s disapproval [8]. Similarly, the stigma associated with conditions like pelvic organ prolapse (POP), sexually transmitted infections, or even a cancer diagnosis can be so powerful that it motivates patients to conceal their symptoms entirely to avoid social judgment [15].

The principal triggers of embarrassment identified across the included studies are summarized in Table 1.

### 3.3. Conceptualizing Medical Embarrassment

To effectively address medical embarrassment, it is crucial to understand that it is not a standalone emotion. The research supports a more nuanced view, distinguishing between different facets of the experience that have distinct triggers and consequences. A primary and clinically significant distinction originally proposed by Consedine, Krivoshekova and Harris in their psychometric validation of the Medical Embarrassment Questionnaire is between bodily embarrassment and judgment concern [18]. Bodily embarrassment pertains directly to the discomfort and anxiety associated with physical exposure—the act of having one’s intimate anatomical areas exposed and touched during an examination. In contrast, judgment concern is a more social and evaluative form of embarrassment, rooted in the fear of being judged negatively for one’s health status, symptoms, or lifestyle choices [18].

This distinction is not merely academic; it has implications for the patient–provider relationship. The recent study by Wu, Pashler and Harris demonstrated that the familiarity a patient has with her doctor affects these two forms of embarrassment in opposing ways [19]. Greater familiarity with a physician tends to mitigate bodily embarrassment, as patients may feel more comfortable with physical exposure in the presence of a known and trusted individual. However, this same familiarity can simultaneously amplify judgment concern [19]. The logic underpinning this paradox consistent with the shame-and-humiliation framework first articulated by Lazare in the medical encounter [2] is that a patient may be more concerned about disappointing or being seen as having failed by a clinician with whom she has an established, positive relationship. Empirical support for the converse effect comes from Hurtaud et al., who reported that an established long-term provider relationship did not, on its own, eliminate unvoiced concerns [4].

This reveals that simply fostering long-term patient relationships, while beneficial for reducing physical discomfort, is not a complete solution. In fact, a strong rapport might make it more difficult for a patient to disclose behaviors she perceives as shameful, such as non-adherence to a treatment plan or engaging in unhealthy habits. This highlights the need for a dual approach to mitigation: one set of strategies must address the physical realities of the exam, while another must focus on creating a communication environment that is explicitly and consistently non-judgmental, regardless of the level of rapport.

Further enriching this understanding, qualitative research conducted in Uganda by Teng et al. introduces a socio-cultural dimension by distinguishing between personal embarrassment and community embarrassment [17]. Personal embarrassment is akin to an internal shyness or discomfort with one’s own genitalia, often linked to a lack of knowledge or the novelty of a procedure. Community embarrassment, on the other hand, is an external, social form of discomfort based on how one might be perceived by others in their community. It is influenced by factors such as the level of privacy in one’s home, personal relationships with local health workers, and community-level stigmas (e.g., confusing an HPV test with an HIV test) [17]. This underscores that embarrassment is not an individual psychological event but is also shaped by the cultural and social context in which care is delivered, suggesting that effective interventions must also consider community-level education and stigma reduction.

### 3.4. Modulating Factors: Patient, Provider, and Systemic Influences

The intensity and impact of embarrassment in the gynecological setting are not uniform across all patients or encounters. A complex interplay of factors related to the patient, the provider, and the healthcare system itself can either exacerbate or mitigate these feelings shaping the patient’s experience and health behaviors [7,12]. The following subsections discuss each in turn, and a structured summary is provided in Table 2.

### 3.5. Patient-Centric Determinants

Several characteristics inherent to the patient influence her vulnerability to embarrassment. Age and life stage are among the most prominent of these factors. Adolescents, in particular, experience a high sense of embarrassment, often stemming from a combination of a lack of knowledge about their bodies and medical procedures, a fear of the unknown, and acute concerns about confidentiality [20,21]. Discussing menstruation, a common reason for a first gynecological visit, can be a major barrier for this age group [20,21]. Evaluation in the adolescent poses several additional challenges to providers, including parent–child–provider reluctance to do a gynecologic history or examination and issues with patient–provider confidentiality, as the parent or guardian is generally involved in the visit and medical decision making [22]. Survey data confirms this vulnerability, with Seamark and Blake reporting that 38% of UK teenagers report feeling embarrassed when seeing a general practitioner, compared to only 16% of women in their fifties. This age group also expresses a much stronger preference for a female doctor, highlighting their increased sensitivity to the dynamics of the clinical encounter [23]. Adolescents are also disproportionately affected by gender-of-provider concerns, as documented in the recent Polish cohort by Henzler et al. [24]. In the reproductive-age middle of the lifespan, the topics that most consistently elicit embarrassment shift toward sexual health, contraception, fertility, and the disclosure of behaviors perceived as socially transgressive [4,8,16]. Background embarrassment may decline somewhat as encounters become more routine, but the impact of embarrassment on disclosure of sensitive topics persists.

At the other end of the lifespan, postmenopausal women face a unique set of challenges. The physiological changes associated with aging and estrogen deficiency, such as vaginal atrophy, can make pelvic examinations physically uncomfortable or even painful, which can compound emotional distress [25]. A dangerous and widespread misconception persists that routine gynecological screenings are no longer necessary after the age of 65, even though current guidelines recommend continued individualized assessment and surveillance—particularly given that the incidence of vulvar cancer rises sharply in this age group [26].

Attitudes toward gynecological care appear to have shifted over the period covered by this review (2000–2025), with greater public discussion of menstruation, menopause, and pelvic floor health, and with media and patient-advocacy initiatives that normalize these topics. The size and uniformity of this secular trend remain uncertain, but its existence is supported by qualitative work in urogynecology populations [27] and should be considered when comparing older and more recent studies.

Beyond demographics, a patient’s psychosocial history and cultural background are critical determinants. A history of sexual abuse or other forms of trauma is associated with heightened anxiety and discomfort during pelvic examinations [28]. For these patients, the exam can be a re-traumatizing experience. Cultural norms also play a powerful role, dictating which topics are considered taboo and shaping attitudes toward help-seeking [14,29]. Williams, Murchie and Bond, in a systematic review of pre-referral visits in gynecological cancer, noted that ethnicity can influence outcomes, with poorer survival rates and a higher number of pre-referral visits observed among some minority women, suggesting that cultural attitudes and potential biases may contribute to delays in care [30]. The broader mixed-methods synthesis by Jouanny et al. similarly identifies stigma and cultural taboo as recurring barriers across heterogeneous urogynecology populations [31].

A patient’s level of knowledge and health literacy is a key factor. Across the included studies, lower health literacy emerges both as a risk factor for embarrassment and as a mediator of its clinical consequences. In the Teng et al. Ugandan ASPIRE cohort, lower knowledge about the purpose of cervical screening was associated with both higher reported embarrassment and lower uptake, and education delivered through peer-led and drama-based programs was associated with measurable decreases in embarrassment over time [17]. In urogynecology, Dunivan et al. and Jouanny et al. similarly identified low awareness of the medical nature of pelvic floor symptoms as a driver of shame, fatalism, and delayed help-seeking [15,31]. A lack of understanding about a condition can breed shame, fear, and a sense of fatalism; the published case report by Butureanu et al. of a virgin patient who eventually presented for a giant ovarian tumor—which could have been diagnosed considerably earlier and with lower surgical risk had embarrassment not delayed presentation—illustrates the clinical consequences at the individual level [32]. Together, these data suggest that educational interventions—both at the individual consultation level and at the community level—are among the most evidence-supported modifiable determinants of embarrassment.

### 3.6. Clinician’s Role

While patient factors establish a baseline of vulnerability, the evidence suggests that clinician behavior during the encounter is consistently identified across the included studies as one of the most influential modifiable determinants of the patient’s experience [28]. Poor communication, characterized by a lack of empathy or clarity, is directly linked to negative patient experiences and increased discomfort [9]. Clinicians who practice with empathy, compassion, and a commitment to open communication create an environment where patients feel safe enough to be truthful and forthcoming with sensitive information [6]. This involves not just a pleasant demeanor but specific skills such as active listening, using clear and non-judgmental language, and taking proactive steps to build trust [14,33]. The negative potential of the clinician’s role is illustrated by the concepts of medical gaslighting and injustice. Medical gaslighting is defined as the act of invalidating a patient’s genuine clinical concern without proper medical evaluation, often due to physician ignorance, bias, or paternalism [34]. This is a manifestation of epistemic injustice, a phenomenon in which a person’s knowledge of their own experience—in this case, their bodily sensations and symptoms—is unfairly discredited or dismissed [35]. Patient accounts describe having their symptoms normalized, trivialized, or dismissed out of hand, leaving them feeling disbelieved, ignored, and even labeled as “crazy” or “depressed” [34]. The recent JAMA Network Open study by Moss et al. quantifies the prevalence of these experiences in vulvovaginal disorders [34], and the AMA Journal of Ethics analysis by Gillespie articulates the ethical framework for response [35]. Such interactions are profoundly harmful: they undermine the therapeutic relationship, cause unjustified suffering, and can lead to life-threatening delays in diagnosis and treatment. This elevates provider dismissal from a matter of poor bedside manner to an act of significant ethical and clinical harm.

The evidence on physician gender is mixed, and reconciling the two strands of literature is important. In the multivariable-adjusted analyses of pelvic examination discomfort reported by Hilden et al., a male gynecologist was **not** significantly associated with increased discomfort [12]. However, when patients are asked about preference and about their comfort with disclosure of sensitive topics—a distinct outcome—a substantial proportion express a preference for a female provider: 45.5% in Yanikkerem et al. [10], with even higher proportions among adolescents in Henzler et al. [24] and in the Saudi Arabian cohort by Alsafar et al. [36]. These two sets of findings are not necessarily contradictory; they reflect different outcomes (discomfort during the examination vs. preference and disclosure comfort) and different patient sub-populations.

The pelvic examination occupies a unique position among medical procedures: the anatomical region examined is also a region invested with sexual and reproductive meaning for both the patient and, often implicitly, the provider. Several included studies [8,24,36] suggest that this symbolic overlap contributes to patient preferences for female providers, particularly when the symptom under discussion is itself perceived as sexual (vaginal discharge, dyspareunia, libido). The clinical implication is not that male providers cannot perform sensitive examinations—the evidence does not support that—but that all providers, irrespective of gender, must actively de-sexualize the encounter through language, draping, and explicit framing of the procedure as a clinical act.

As previously discussed, familiarity with the provider presents a complex dynamic, decreasing bodily embarrassment while potentially increasing judgment concern [18,19]. It is also important to recognize that providers are not immune to discomfort. A clinician’s own personal discomfort with sensitive topics, perceived time constraints, or fear of causing offense can lead them to avoid initiating these crucial conversations, thereby perpetuating a cycle of silence [4].

**Table 2:** Modulating factors influencing patient embarrassment.

Factor Type	Specific Factor	Effect on Embarrassment (Increase/Decrease) & Rationale
Patient-Related	Age (Adolescent)	Increases embarrassment and shows a strong preference for female provider [23,24].
	Age (Postmenopausal)	Physical discomfort from atrophy can increase distress; misconceptions may lead to avoidance [25,26].
	History of Trauma/Abuse	Strongly associated with increased discomfort and anxiety during exams [28].
	Cultural Norms/Ethnicity	Can increase embarrassment for “taboo” topics; may influence help-seeking delays [14,30,31].
	Lack of Knowledge	Increases shame and fear; education can decrease embarrassment over time [15,17,31].
Provider-Related	Communication Style	Empathetic, open communication decreases embarrassment; poor communication increases it [6].
	Medical Gaslighting/Dismissal	Dramatically increases distress, erodes trust, and causes psychological harm [34,35].
	Provider Gender	Many patients report increased embarrassment and prefer a female provider [10,24,36].
	Provider Familiarity	Decreases bodily embarrassment but can increase judgment-concern embarrassment [18,19].
	Provider’s Own Discomfort	Can lead providers to avoid sensitive topics, indirectly sustaining patient embarrassment [4].
Systemic/Environmental	Clinic Environment	Dim lighting and music can decrease embarrassment [37].
	Privacy	Lack of privacy in dwellings or clinic setting increases community embarrassment [17].
	Presence of Chaperone/Support	Offering a chaperone or support person can decrease anxiety and provide a sense of safety [6].
	Presence of Others (Family/Student)	Presence of a family member (69%) or student (43%) is seen as an obstacle to discussing embarrassing issues [4].

### 3.7. The Impact on Help-Seeking

The internal experience of embarrassment and shame does not remain contained within the patient’s psyche; it translates directly into a set of observable behaviors that systematically undermine the process of receiving medical care. These behaviors—avoidance of care, concealment of information, and non-adherence to treatment—are not signs of patient apathy or negligence. Rather, they represent active, albeit counterproductive, coping mechanisms employed by the patient to manage the acute emotional threat of shame and humiliation [1]. While these actions may provide short-term emotional relief by avoiding a feared situation, their long-term clinical consequences can be substantial.

One of the most significant behavioral consequences of embarrassment is the delay or complete avoidance of necessary medical care. Embarrassment is cited as a primary reason why women fail to participate in essential preventive screenings for conditions like sexually transmitted infections (STIs) and cervical cancer [17,29]. The scope of this avoidance is broad; for example, in the survey reported by Wu et al., more than half of all adults had been deterred from seeking medical attention for potentially serious symptoms specifically due to the fear of embarrassment [19]. This pattern of delay is prevalent across a wide range of gynecological conditions. Women with symptoms of pelvic floor dysfunction, such as urinary incontinence, often delay seeking help for years. In the cohort reported by Adelowo et al. over 60% of women waited at least a year after symptom onset before their initial visit [38]. Women experiencing symptoms suggestive of gynecological cancer may delay presentation due to the intimate nature of the symptoms and the associated embarrassment [30]. This delay is often facilitated by a process of normalization, where patients convince themselves that their symptoms—such as heavy menstrual bleeding or urinary leakage—are simply a normal part of being a woman or aging and are something to be endured in silence [15]. Unmarried women may feel particularly stigmatized and delay seeking reproductive health services until their symptoms become unbearable, specifically to avoid the shame of being in a clinical setting they associate with married individuals [29]. This active avoidance, driven by a desire to protect oneself from shame, creates a dangerous gap between the onset of symptoms and the initiation of medical care.

### 3.8. Information During the Consultation

Even when a patient overcomes the initial barrier of embarrassment to schedule an appointment, the emotion continues to exert a powerful influence within the consultation room. A substantial number of patients engage in information concealment, intentionally withholding medically relevant details from their clinicians. Overall, 32.3% of patients in the Hurtaud et al. study admitted to having unspoken concerns during a visit due to embarrassment or fear of judgment [4]. The reasons for this concealment are rooted in the same fears that drive care avoidance: patients worry about being judged, lectured, or stigmatized for their behaviors or symptoms [14]. This can manifest as a doorknob disclosure, where a patient postpones voicing her true, most embarrassing concern until the very end of the consultation, often as she is about to leave [16]. This timing leaves insufficient opportunity for the clinician to adequately address the issue, effectively rendering the disclosure useless for that visit. The direct consequence of this information withholding is a compromised diagnostic process. Clinicians are forced to make critical decisions based on an incomplete and potentially misleading clinical picture, which can lead to misdiagnosis, the initiation of inappropriate treatments, or the failure to provide necessary care [14]. The patient’s silence, intended as a shield against emotional discomfort, becomes a direct threat to her physical well-being.

### 3.9. Non-Adherence to Medical Advice and Treatment

The impact of embarrassment extends beyond the initial consultation and diagnosis to affect a patient’s adherence to recommended screenings and treatments. The negative emotional symptoms provoked by the pelvic exam itself—including pain, anxiety, and embarrassment—can directly interfere with a patient’s compliance with follow-up appointments and preventive health screenings [11]. The connection between the emotional experience and adherence is quantifiable. For instance, the perception that a Pap test is painful, a feeling deeply intertwined with the anxiety and embarrassment of the procedure, has been associated with a nearly five-fold increase in the risk of non-adherence to cervical cancer screening recommendations in the cohort by Hoyo et al. [39]. HPV self-sampling has been explored as one strategy to mitigate this barrier [40]. For women being treated for conditions like vulvovaginal candidosis (VVC), the shame associated with the condition can be so significant that it leads them to drop out of treatment studies or avoid necessary follow-up care as documented in the qualitative study by Erfaninejad et al. [41]. While the links are sometimes less direct, the factors that inhibit the initial consultation—shame, fear, lack of knowledge—are also implicated in poor adherence to long-term treatment regimens for chronic conditions like polycystic ovary syndrome (PCOS) [42,43]. By creating a negative association with the entire process of gynecological care, embarrassment severely diminishes patient engagement and compliance, leading to a breakdown in the therapeutic plan and a failure to achieve desired health outcomes.

### 3.10. Health and Economic Consequences of Suboptimal Care

The behavioral manifestations of embarrassment—care avoidance, information concealment, and non-adherence—are not benign. They initiate a number of events that results in significant and measurable negative consequences for patient health [3,30], healthcare system efficiency, and the patient–provider relationship itself [44]. The failure to address patient embarrassment leads directly to delayed diagnoses, which in turn drives up healthcare costs and contributes to poorer clinical outcomes.

The link between embarrassment-driven behavior and delayed diagnosis is well-established across several gynecological conditions, with endometriosis serving as a particularly relevant case study. This delay is not a static waiting period; it is a period during which the disease can progress. Consequently, a longer diagnostic delay is directly associated with a worsening of the condition and a more advanced stage of disease at the time of diagnosis [3]. This pattern is not unique to endometriosis. For cervical and vulvar cancers, delays in presentation have been associated with more advanced FIGO stage at diagnosis, reduced eligibility for fertility-sparing or organ-preserving surgery, and lower 5-year survival [30]. Observed disparities in survival rates among different ethnic populations may be linked, in part, to cultural attitudes that foster embarrassment and delay presentation [30]. Similarly, in the case of pelvic floor dysfunction, women who postpone seeking care due to shame are more likely to present with increased symptom severity when they finally do consult a physician [31,38]. These conditions, while not typically life-threatening, have a severe impact on quality of life, contributing to higher rates of depression and anxiety [38]. In all these cases, embarrassment acts as a catalyst for disease progression, allowing conditions to become more severe and complex before they are ever addressed.

The clinical consequences of delayed diagnosis are mirrored by a significant economic burden. Delays caused by embarrassment often lead to more complex conditions that are harder and more expensive to treat, transforming what might have been a manageable, low-cost issue into a complex and expensive medical problem. The data on endometriosis provides a clear quantitative illustration of this effect. Studies have shown that longer diagnostic delays are associated with substantially higher pre-diagnosis healthcare costs. Patients with a long delay (over 3 years) incurred average pre-diagnosis costs of $34,460, compared to $21,489 for patients with a short delay (less than 1 year) [44]. This increase in cost is driven by a shift in healthcare utilization. Instead of addressing symptoms in a routine, low-acuity primary care or gynecology setting, patients with delayed presentations are more likely to access the healthcare system through more expensive and inefficient channels. The higher costs are directly linked to a greater number of emergency room visits and inpatient hospitalizations during the pre-diagnosis period [44]. The initial avoidance of a routine consultation, motivated by embarrassment, ultimately results in the need for high-acuity, high-cost interventions to manage a more advanced condition. Furthermore, the practice of withholding information during a consultation can lead to unnecessary and expensive investigative tests, as clinicians attempt to diagnose a problem without having all the relevant facts [32]. This demonstrates that investing in strategies to mitigate patient embarrassment is not merely a matter of improving patient experience; it is a fiscally responsible public health strategy that can reduce downstream costs and promote more efficient use of healthcare resources.

### 3.11. Trust and Patient Safety

Beyond the direct clinical and economic impacts, embarrassment and the provider behaviors that exacerbate it cause a fundamental erosion of trust in the patient–provider relationship, with serious implications for patient safety. Dismissive behaviors and medical gaslighting are profoundly harmful, invalidating the patient’s experience and undermining her willingness to seek or trust medical care in the future [34,35]. This breakdown in trust is a critical patient safety issue. Poor communication, which is both a cause and a consequence of an environment fraught with embarrassment, is a major contributor to medical errors. Studies have indicated that suboptimal communication may be responsible for up to 80% of all preventable adverse events (pAEs) in clinical settings [5]. The field of gynecology is not immune to this problem; one meta-analysis found the incidence of adverse events in gynecological hospital admissions to be 10.8%, with over half of those events being preventable [5]. When patients feel too embarrassed to ask questions or withhold critical information about their symptoms or history, the risk of misdiagnosis and inappropriate treatment rises dramatically, directly compromising their safety and well-being [14].

## 4. Discussions

### 4.1. Strategies for Patient-Centered Gynecological Practice

The included studies collectively suggest that embarrassment is modifiable through three complementary levels of intervention—communication, the procedural and physical environment, and system-level training and education [5,45,46]. The most effective mitigation strategies are those that are proactive, patient-empowering, and systemic. They shift the responsibility for addressing embarrassment from the patient to the healthcare provider and system. Addressing this issue will require changes at multiple levels, including communication practices, clinical environments, and provider training.

### 4.2. Transforming the Communication and Trust

The core of any effective mitigation strategy lies in revolutionizing communication within the clinical encounter. A foundational step is the widespread adoption of Trauma-Informed Care. This approach requires clinicians to operate from the assumption that any patient may have a history of trauma and to design the interaction to foster a sense of physical and psychological safety, control, and empowerment [33]. This is not a specialized technique for a subset of patients but a universal precaution that benefits everyone by creating a more respectful and sensitive care environment. Building on this foundation, clinicians must master specific patient-centered communication techniques. A critical element is the proactive initiation of sensitive conversations. In the Hurtaud et al. French primary-care survey (n = 480 patients with previously unvoiced concerns), a remarkable 78% of patients report that they would feel more at ease if their doctor raised the difficult or embarrassing topic first [4]. This simple act removes the burden of initiation from the patient and signals that the topic is normal, acceptable, and important to discuss. This should be paired with a commitment to normalize and educate. Clinicians can actively reduce shame by explaining the commonality of concerns like abnormal vaginal discharge or incontinence, educating the patient about the purpose of each step of the exam, and providing clear information about their condition [13]. The evidence base for education as a mitigation strategy distinguishes between (i) observational data and (ii) intervention data. Observational data consistently link higher knowledge to lower embarrassment, with the strongest examples coming from the Ugandan ASPIRE cohort [17] and from the urogynecology literature [15,31]. Intervention data remain more limited and heterogeneous: the Lippke et al. communication-intervention protocol [5] and the Derksen et al. HAPA-based psychological intervention [46] provide the strongest available trial-level support, but additional well-designed trials are needed. These techniques are most effective within a framework of trust and rapport, built through active listening, the use of open-ended questions, and the consistent validation of the patient’s feelings and concerns [6]. Explicitly reaffirming the confidentiality of the consultation can also be highly effective, helping nearly half of all patients feel more comfortable being forthcoming [4]. The entire interaction should be guided by the principles of shared decision-making. This represents a move away from a paternalistic model toward a collaborative partnership where the patient is an active participant in her own care. This includes thoroughly discussing expectations of pain and the available options for pain management before a potentially uncomfortable procedure begins, giving the patient agency and control [47].

Earlier sections of this review noted that the presence or role of a nurse or chaperone is consistently identified by patients as a factor that can ease the discomfort of a gynecological encounter. This deserves explicit integration into the discussion of patient-centered strategies. In the Yanikkerem cohort, 71.8% of patients expected the attending nurse to demonstrate understanding and gentleness during the pelvic examination, and a further 28.2% expected the nurse to provide procedural information [10]. The American College Health Association’s best-practice guidance on sensitive examinations [45] recommends that every institution have a clear, written policy on chaperone use, that chaperones be trained, and that the chaperone option be proactively offered rather than only offered on request. Proactive offering—i.e., the clinician saying explicitly “I will ask a chaperone to be present unless you would prefer not”—both normalizes the chaperone’s presence and removes the implicit burden on the patient to negotiate it.

### 4.3. Optimizing the Physical Environment and Procedure

In parallel with improving communication, optimizing the physical environment and the procedures themselves can significantly reduce patient discomfort and anxiety. A key principle is to enhance patient comfort and control during the examination. This can be achieved through several practical measures. Offering the use of smaller or warmed speculums can reduce physical discomfort [9]. Ensuring private facilities for undressing and using sensitive and thoughtful draping techniques can mitigate feelings of exposure [6]. Clinicians should also consider offering alternative examination positions: the standard lithotomy position can feel particularly vulnerable, and alternative positions, such as side-lying or frog-leg, can reduce both physical discomfort and psychological anxiety, especially for patients with a history of trauma or chronic pain [33]. Perhaps most importantly, providers must explicitly and repeatedly communicate to the patient that she is in control. Stating clearly that she can ask to pause or stop the examination at any time is a powerful way to return a sense of agency to the patient, which can dramatically reduce her anxiety [6].

The ambient environment of the examination room can also be modified to promote relaxation. Simple, low-cost interventions have been shown to be effective. One randomized controlled trial found that simply dimming the lights and playing light instrumental music during a procedure resulted in a clinically significant decrease in patient-reported embarrassment scores [37]. Creating a generally welcoming and age-appropriate clinic environment can also contribute to a more positive overall experience [48].

### 4.4. High-Level Interventions

While individual clinicians can make a difference, lasting change requires broader, system-level interventions that address the root causes of embarrassment and equip the entire healthcare system to provide more sensitive care. Patient education and public health campaigns are essential for tackling societal-level stigma and lack of knowledge. Strategies such as peer-to-peer education programs, community-based drama performances, and targeted media campaigns can help overcome psychosocial barriers, normalizing intimate health concerns are also documented in qualitative work in urogynecology populations [27]. The proliferation of websites and media initiatives aimed at normalizing intimate health concerns can also empower patients to seek care [17]. There must also be a renewed focus on provider training. Medical education curricula must incorporate formal training, direct observation, and structured feedback on conducting sensitive examinations and on navigating informed-consent conversations. The American College Health Association’s best-practice document [45], the AMA Journal of Ethics analysis by Hillard [49], and the recent OSCE-based study by Alsubaie et al. [50] all describe concrete training modalities. This training must go beyond technical skills to explicitly teach and assess competencies in empathy, patient-centered communication, and trauma-informed care. Furthermore, training programs should include modules on implicit bias and epistemic humility to help providers recognize and prevent the dismissal of patient concerns that constitutes medical gaslighting [6,35]. By embedding these principles early and consistently throughout medical training, the healthcare system can cultivate clinicians who are inherently equipped to create a safer and more dignifying care experience for all patients. These strategies are summarized in Table 3.

This review has several limitations that should be considered when interpreting its findings. First, the included studies are heterogeneous in design, population, and outcome measures, which limits the ability to draw direct comparisons or establish causality. Many studies rely on self-reported data, which may be subject to recall bias and social desirability bias, particularly given the sensitive nature of the topic.

Second, there is no universally accepted definition or standardized measurement of embarrassment in medical settings, resulting in variability in how this construct is operationalized across studies. The Medical Embarrassment Questionnaire [18] is one of the few validated instruments, but it is not used uniformly. This lack of standardization may affect the consistency and generalizability of reported findings.

Third, publication bias may be present, as studies demonstrating significant psychosocial barriers or negative outcomes are more likely to be published. Additionally, the majority of available data originates from high-income settings, which may limit the applicability of findings to low- and middle-income contexts where cultural norms and healthcare systems differ.

Finally, as a narrative synthesis of the existing literature, this review is limited by the quality and scope of the included studies and does not provide quantitative pooled estimates. Future research using standardized definitions, prospective designs, and diverse populations is needed to better characterize the impact of embarrassment on gynecological care and to evaluate targeted interventions.

## 5. Conclusions

The synthesized evidence indicates that patient embarrassment is a quantifiable barrier to effective gynecological care. It functions as an affective determinant of health, leading to behavioral responses such as care avoidance, information concealment, and treatment non-adherence. These behaviors contribute to delayed diagnoses, poorer health outcomes, and an increased economic burden on the healthcare system. The link between the emotional experience of shame and adverse clinical outcomes is well documented. However, this barrier is modifiable. Research demonstrates that patient embarrassment can be mitigated by shifting responsibility from the patient to the healthcare system. Clinicians, educators, and administrators must proactively create clinical environments and practices that prioritize safety and respect. Implementing trauma-informed principles, patient-centered communication, and procedural modifications that prioritize patient comfort and agency are essential clinical practices. This review highlights the need for systemic changes in gynecological practice. Clinicians require training to manage psychosocial needs, and medical educators should incorporate empathy, trust-building, and sensitive communication into curricula. Administrators must design environments that protect privacy and dignity. Recognizing embarrassment as a clinical variable and implementing mitigation strategies can reduce this barrier to care. Prioritizing psychological safety is necessary to maintain the patient–provider relationship and improve health outcomes for women.

## Figures and Tables

**Table 1:** Primary triggers of embarrassment in the gynecological setting.

Category	Specific Trigger/Question	Patient Experience/Clinical Context
Physical/Procedural	Undressing [9]	
	Speculum Insertion [11]	Provokes “pain, discomfort, anxiety, fear, embarrassment, and irritability”.
	Physical Exposure of Genitalia [9]	Leads to feelings of “lost dignity and shyness” and being in a “vulnerable situation with loss of control”.
	Lack of Gentleness [7]	Physical unpleasantness from a “cold instrument and the lack of gentleness”.
Communicative/Topical	Sexual History/Behavior [4,16]	Main motive for unvoiced concerns: “sexual behavior, libido… vaginal dryness”.
	Unprotected Sex [8]	“It’s the whole embarrassment, the shame because you made a mistake you know”.
	“Taboo” Symptoms [13]	Vaginal odor, abnormal discharge, leaking urine, lumps/bumps.
	Mental Health [4]	Second most common unvoiced topic: “psychological disorders, low mood, anxiety”.
Psychological/Social	Stigma of Diagnosis [17]	Stigma associated with cervical cancer or STIs acts as a psychosocial barrier.
	Fear of Judgment [4,18]	A primary reason for unvoiced concerns is a “fear of being judged”.
	Fear of Abnormal Findings [19]	Driven by a “fear of discovery of a pathological condition”.
	Concerns about Cleanliness [9]	Centers on “worries about cleanliness, qualms about vaginal odor”.

**Table 3:** Multi-level strategies for mitigating patient embarrassment strategy domain.

	Specific Intervention	Rationale/Mechanism of Action
Communication	Proactively initiate sensitive topics [4]	Reduces patient’s burden of initiation; signals that the topic is normal and acceptable to discuss, normalizing the conversation.
	Adopt Trauma-Informed Care as a universal precaution [33]	Creates a culture of physical and psychological safety; equips providers to manage patient distress and avoid re-traumatization.
	Use explicit, empathetic communication and shared decision-making [6,47]	Builds trust, validates patient experience, and returns a sense of agency and control to the patient, reducing feelings of powerlessness.
	Normalize common symptoms and educate the patient [13,17]	Reduces shame by reframing symptoms as medical issues rather than personal failings; knowledge diminishes fear of the unknown.
Procedural/Environmental	Offer alternative exam positions (e.g., side-lying) [33]	Increases patient’s sense of control and physical comfort; reduces feelings of vulnerability associated with the lithotomy position.
	Enhance physical comfort (e.g., warmed speculum, sensitive draping) [6,9]	Reduces negative physical stimuli that can exacerbate psychological distress and anxiety.
	Modify the ambient environment (e.g., dim lighting, music) [37]	Creates a more relaxing and private atmosphere, which has been shown to directly reduce patient-reported embarrassment scores.
	Offer a chaperone or support person [10,45]	Provides a sense of safety, balances the power dynamic in the room, and offers emotional support to an anxious patient.
Systemic	Implement provider training on sensitive exams and communication [45,49,50]	Equips clinicians with the necessary skills to prevent and manage patient embarrassment, making sensitive care a core competency.
	Launch public health campaigns to normalize gynecological health [35]	Reduces societal-level stigma and increases knowledge, which are root causes of patient embarrassment and care avoidance.

## Data Availability

No new data were created or analyzed in this study.

## Abbreviations

pAEs	preventable adverse events
POP	pelvic organ prolapse
